# Physical Activity and Incident Obesity Across the Spectrum of Genetic Risk for Obesity

**DOI:** 10.1001/jamanetworkopen.2024.3821

**Published:** 2024-03-27

**Authors:** Evan L. Brittain, Lide Han, Jeffrey Annis, Hiral Master, Andrew Hughes, Dan M. Roden, Paul A. Harris, Douglas M. Ruderfer

**Affiliations:** 1 Division of Cardiovascular Medicine, Vanderbilt University Medical Center, Nashville, Tennessee; 2 Center for Digital Genomic Medicine, Department of Medicine, Vanderbilt University Medical Center, Nashville, Tennessee; 3 Division of Genetic Medicine, Vanderbilt Genetics Institute, Vanderbilt University Medical Center, Nashville, Tennessee; 4 Vanderbilt Institute of Clinical and Translational Research, Vanderbilt University Medical Center, Nashville, Tennessee; 5 Department of Medicine, Vanderbilt University Medical Center, Nashville, Tennessee; 6 Department of Pharmacology, Vanderbilt University Medical Center, Nashville, Tennessee; 7 Department of Biomedical Informatics, Vanderbilt University Medical Center, Nashville, Tennessee; 8 Department of Biomedical Engineering, Vanderbilt University Medical Center, Nashville, Tennessee; 9 Department of Biostatistics, Vanderbilt University Medical Center, Nashville, Tennessee; 10 Department of Psychiatry and Behavioral Sciences, Vanderbilt University Medical Center, Nashville, Tennessee

## Abstract

**Importance:**

Despite consistent public health recommendations, obesity rates in the US continue to increase. Physical activity recommendations do not account for individual genetic variability, increasing risk of obesity.

**Objective:**

To use activity, clinical, and genetic data from the All of Us Research Program (AoURP) to explore the association of genetic risk of higher body mass index (BMI) with the level of physical activity needed to reduce incident obesity.

**Design, Setting, and Participants:**

In this US population–based retrospective cohort study, participants were enrolled in the AoURP between May 1, 2018, and July 1, 2022. Enrollees in the AoURP who were of European ancestry, owned a personal activity tracking device, and did not have obesity up to 6 months into activity tracking were included in the analysis.

**Exposure:**

Physical activity expressed as daily step counts and a polygenic risk score (PRS) for BMI, calculated as weight in kilograms divided by height in meters squared.

**Main Outcome and Measures:**

Incident obesity (BMI ≥30).

**Results:**

A total of 3124 participants met inclusion criteria. Among 3051 participants with available data, 2216 (73%) were women, and the median age was 52.7 (IQR, 36.4-62.8) years. The total cohort of 3124 participants walked a median of 8326 (IQR, 6499-10 389) steps/d over a median of 5.4 (IQR, 3.4-7.0) years of personal activity tracking. The incidence of obesity over the study period increased from 13% (101 of 781) to 43% (335 of 781) in the lowest and highest PRS quartiles, respectively (*P* = 1.0 × 10^−20^). The BMI PRS demonstrated an 81% increase in obesity risk (*P* = 3.57 × 10^−20^) while mean step count demonstrated a 43% reduction (*P* = 5.30 × 10^−12^) when comparing the 75th and 25th percentiles, respectively. Individuals with a PRS in the 75th percentile would need to walk a mean of 2280 (95% CI, 1680-3310) more steps per day (11 020 total) than those at the 50th percentile to have a comparable risk of obesity. To have a comparable risk of obesity to individuals at the 25th percentile of PRS, those at the 75th percentile with a baseline BMI of 22 would need to walk an additional 3460 steps/d; with a baseline BMI of 24, an additional 4430 steps/d; with a baseline BMI of 26, an additional 5380 steps/d; and with a baseline BMI of 28, an additional 6350 steps/d.

**Conclusions and Relevance:**

In this cohort study, the association between daily step count and obesity risk across genetic background and baseline BMI were quantified. Population-based recommendations may underestimate physical activity needed to prevent obesity among those at high genetic risk.

## Key Points

**Question:**

Does the degree of physical activity associated with incident obesity vary by genetic risk?

**Findings:**

In this cohort study of 3124 adults, individuals at high genetic risk of obesity needed higher daily step counts to reduce the risk of obesity than those at moderate or low genetic risk.

**Meaning:**

These findings suggest that individualized physical activity recommendations that incorporate genetic background may reduce obesity risk.

## Teaser

This cohort study uses longitudinal activity monitoring and genome sequencing data from the All of Us Research Program to quantify the combined association of genetic risk for body mass index and physical activity with the risk of incident obesity in US adults.

## Introduction

In 2000, the World Health Organization declared obesity the greatest threat to the health of Westernized nations.^1^ In the US, obesity accounts for over 400 000 deaths per year and affects nearly 40% of the adult population. Despite the modifiable nature of obesity through diet, exercise, and pharmacotherapy, rates have continued to increase.

Physical activity recommendations are a crucial component of public health guidelines for maintaining a healthy weight, with increased physical activity being associated with a reduced risk of obesity.^2,3,4^ Fitness trackers and wearable devices have provided an objective means to capture physical activity, and their use may be associated with weight loss.^5^ Prior work leveraging these devices has suggested that taking around 8000 steps/d substantially mitigates risk of obesity.^3,4^ However, current recommendations around physical activity do not take into account other contributors such as caloric intake, energy expenditure, or genetic background, likely leading to less effective prevention of obesity for many people.^6^

Obesity has a substantial genetic contribution, with heritability estimates ranging from 40% to 70%.^7,8^ Prior studies^9,10,11^ have shown an inverse association between genetic risk and physical activity with obesity, whereby increasing physical activity can help mitigate higher genetic risk for obesity. These results have implications for physical activity recommendations on an individual level. Most of the prior work^9,10,11^ focused on a narrow set of obesity-associated variants or genes and relied on self-reported physical activity, and more recent work using wearable devices has been limited to 7 days of physical activity measurements.^12^ Longer-term capture in large populations will be required to accurately estimate differences in physical activity needed to prevent incident obesity.

We used longitudinal activity monitoring and genome sequencing data from the All of Us Research Program (AoURP) to quantify the combined association of genetic risk for body mass index (BMI; calculated as weight in kilograms divided by height in meters squared) and physical activity with the risk of incident obesity. Activity monitoring was quantified as daily step counts obtained from fitness tracking devices. Genetic risk was quantified by using a polygenic risk score (PRS) from a large-scale genomewide association study (GWAS) of BMI.^13^ We quantified the mean daily step count needed to overcome genetic risk for increased BMI. These findings represent an initial step toward personalized exercise recommendations that integrate genetic information.

## Methods

### Cohort Description

Details on the design and execution of the AoURP have been published previously.^14^ The present study used AoURP Controlled Tier dataset, version 7 (C2022Q4R9), with data from participants enrolled between May 1, 2018, and July 1, 2022. Participants who provided informed consent could share data from their own activity tracking devices from the time their accounts were first created, which may precede the enrollment date in AoURP. We followed the Strengthening the Reporting of Observational Studies in Epidemiology (STROBE) reporting guideline. In this study, only the authorized authors who completed All of Us Responsible Conduct of Research training accessed the deidentified data from the Researcher Workbench (a secured cloud-based platform). Since the authors were not directly involved with the participants, institutional review board review was exempted in compliance with AoURP policy.

Activity tracking data for this study came from the Bring Your Own Device program that allowed individuals who already owned a tracking device (Fitbit, Inc) to consent to link their activity data with other data in the AoURP. By registering their personal device on the AoURP patient portal, patients could share all activity data collected since the creation of their personal device account. For many participants, this allowed us to examine fitness activity data collected prior to enrollment in the AoURP. Activity data in AoURP are reported as daily step counts. We excluded days with fewer than 10 hours of wear time to enrich our cohort for individuals with consistently high wear time. The initial personal activity device cohort consisted of 12 766 individuals. Consistent with our prior data curation approach, days with less than 10 hours of wear time, less than 100 steps, or greater than 45 000 steps or for which the participant was younger than 18 years were removed. For time-varying analyses, mean daily steps were calculated on a monthly basis for each participant. Months with fewer than 15 valid days of monitoring were removed.

The analytic cohort included only individuals with a BMI of less than 30 at the time activity monitoring began. The primary outcome was incident obesity, defined as a BMI of 30 or greater documented in the medical record at least 6 months after initiation of activity monitoring. The latter stipulation reduced the likelihood that having obesity predated the beginning of monitoring but had not yet been clinically documented. We extracted BMI values and clinical characteristics from longitudinal electronic health records (EHRs) for the consenting participants who were associated with a health care provider organization funded by the AoURP. The EHR data have been standardized using the Observational Medical Outcomes Partnership Common Data Model.^15^ In the AoURP, upon consent, participants are asked to complete the Basics survey, in which they may self-report demographic characteristics such as race, ethnicity, and sex at birth.

### Genome Sequencing Quality Control and Filtering

We filtered the data to include only biallelic, autosomal single-nucleotide variants (SNVs) that had passed AoURP initial quality control.^16^ We then removed duplicate-position SNVs and kept only individual genotypes with a genotype quality greater than 20. We further filtered the SNVs based on their Hardy-Weinberg equilibrium *P* value (>1.0 × 10^−15^) and missing rate (<5%) across all samples. Next, we divided the samples into 6 groups (Admixed American, African, East Asian, European, Middle Eastern, and South Asian) based on their estimated ancestral populations^16,17^ and further filtered the SNVs within each population based on minor allele frequency (MAF) (>0.01), missing rate (<0.02), and Hardy-Weinberg equilibrium *P* value (>1.0 × 10^−6^). The SNVs were mapped from Genome Reference Consortium Human Build 38 with coordinates to Build 37. Because the existing PRS models have limited transferability across ancestry groups and to ensure appropriate power of the subsequent PRS analysis, we limited our analysis to the populations who had a sample size of greater than 500, resulting in 5964 participants of European ancestry with 5 515 802 common SNVs for analysis.

To generate principal components, we excluded the regions with high linkage disequilibrium, including chr5:44-51.5 megabase (Mb), chr6:25-33.5 Mb, chr8:8-12 Mb, and chr11:45-57 Mb. We then pruned the remaining SNVs using PLINK, version 1.9 (Harvard University), pairwise independence function with 1-kilobase window shifted by 50 base pairs and requiring *r*^2^ < 0.05 between any pair, resulting in 100 983 SNPs for further analysis.^18^ Principal component analysis was run using PLINK, version 1.9. The European ancestry linkage disequilibrium reference panel from the 1000 Genomes Project phase 3 was downloaded, and nonambiguous SNPs with MAF greater than 0.01 were kept in the largest European ancestry GWAS summary statistics of BMI.^13^ We manually harmonized the strand-flipping SNPs among the SNP information file, GWAS summary statistics files, and the European ancestry PLINK extended map files (.bim).

We used PRS–continuous shrinkage to infer posterior SNP effect sizes under continuous shrinkage priors with a scaling parameter set to 0.01, reflecting the polygenic architecture of BMI. GWAS summary statistics of BMI measured in 681 275 individuals of European ancestry was used to estimate the SNP weights.^19^ The scoring command in PLINK, version 1.9, was used to produce the genomewide scores of the AoURP European individuals with their quality-controlled SNP genotype data and these derived SNP weights.^20^ Finally, by using the genomewide scores as the dependent variable and the 10 principal components as the independent variable, we performed linear regression, and the obtained residuals were kept for the subsequent analysis. To check the performance of the PRS estimate, we first fit a generalized regression model with obesity status as the dependent variable and the PRS as the independent variable with age, sex, and the top 10 principal components of genetic ancestry as covariates. We then built a subset logistic regression model, which only uses the same set of covariates. By comparing the full model with the subset model, we measured the incremental Nagelkerke *R*^2^ value to quantify how much variance in obesity status was explained by the PRS.

### Statistical Analysis

Differences in clinical characteristics across PRS quartiles were assessed using the Wilcoxon rank sum or Kruskal-Wallis test for continuous variables and the Pearson χ^2^ test for categorical variables. Cox proportional hazards regression models were used to examine the association among daily step count (considered as a time-varying variable), PRS, and the time to event for obesity, adjusting for age, sex, mean baseline step counts, cancer status, coronary artery disease status, systolic blood pressure, alcohol use, educational level, and interaction term of PRS × mean steps. We presented these results stratified by baseline BMI and provided a model including baseline BMI in eFigure 2 in Supplement 1 as a secondary analysis due to collinearity between BMI and PRS.

Cox proportional hazards regression models were fit on a multiply imputed dataset. Multiple imputation was performed for baseline BMI, alcohol use, educational status, systolic blood pressure, and smoking status using bootstrap and predictive mean matching with the aregImpute function in the Hmisc package of R, version 4.2.2 (R Project for Statistical Computing). Continuous variables were modeled as restricted cubic splines with 3 knots, unless the nonlinear term was not significant, in which case it was modeled as a linear term. Fits and predictions of the Cox proportional hazards regression models were obtained using the rms package in R, version 4.2.2. The Cox proportional hazards regression assumptions were checked using the cox.zph function from the survival package in R, version 4.2.2.

To identify the combinations of PRS and mean daily step counts associated with a hazard ratio (HR) of 1.00, we used a 100-knot spline function to fit the Cox proportional hazards regression ratio model estimations across a range of mean daily step counts for each PRS percentile. We then computed the inverse of the fitted spline function to determine the mean daily step count where the HR equals 1.00 for each PRS percentile. We repeated this process for multiple PRS percentiles to generate a plot of mean daily step counts as a function of PRS percentiles where the HR was 1.00. To estimate the uncertainty around these estimations, we applied a similar spline function to the upper and lower estimated 95% CIs of the Cox proportional hazards regression model to find the 95% CIs for the estimated mean daily step counts at each PRS percentile. Two-sided *P* < .05 indicated statistical significance.

## Results

We identified 3124 participants of European ancestry without obesity at baseline who agreed to link their personal activity data and EHR data and had available genome sequencing. Among those with available data, 2216 of 3051 (73%) were women and 835 of 3051 (27%) were men, and the median age was 52.7 (IQR, 36.4-62.8) years. In terms of race and ethnicity, 2958 participants (95%) were White compared with 141 participants (5%) who were of other race or ethnicity (which may include Asian, Black or African American, Middle Eastern or North African, Native Hawaiian or Other Pacific Islander, multiple races or ethnicities, and unknown race or ethnicity) (Table). The analytic sample was restricted to individuals assigned European ancestry based on the All of Us Genomic Research Data Quality Report.^16^ A study flowchart detailing the creation of the analytic dataset is provided in eFigure 1 in Supplement 1. The BMI-based PRS explained 8.3% of the phenotypic variation in obesity (β = 1.76; *P* = 2 × 10^−16^). The median follow-up time was 5.4 (IQR, 3.4-7.0) years and participants walked a median of 8326 (IQR, 6499-10 389) steps/d. The incidence of obesity over the study period was 13% (101 of 781 participants) in the lowest PRS quartile and 43% (335 of 781 participants) in the highest PRS quartile (*P* = 1.0 × 10^−20^). We observed a decrease in median daily steps when moving from lowest (8599 [IQR, 6751-10 768]) to highest (8115 [IQR, 6340-10 187]) PRS quartile (*P* = .01).

**Table.** Participant Characteristics Across PRS Quartiles

Characteristic	PRS quartile^a^				*P* value^b^
	1 (n = 781)	2 (n = 781)	3 (n = 781)	4 (n = 781)	
Age, median (IQR), y	54.2 (38.5-63.3)	52.9 (35.7-63.0)	53.6 (37.9-64.0)	49.8 (34.0-60.9)	3.0 × 10^−4^
Self-reported race					
White	745/778 (96)	731/771 (95)	744/776 (96)	738/774 (95)	.74
Other or missing^c^	33/778 (4)	40/771 (5)	32/776 (4)	36/774 (5)	
Sex					
Women	544/764 (71)	559/766 (73)	573/761 (75)	540/760 (71)	.21
Men	220/764 (29)	207/766 (27)	188/761 (25)	220/760 (29)	
BMI, median (IQR)	24.0 (21.8-25.8)	24.5 (22.7-26.5)	25.0 (23.0-27.1)	25.5 (23.8-27.3)	1.11 × 10^−16^
Educational level					
College degree	625/763 (82)	610/764 (80)	582/757 (77)	556/754 (74)	.005
No college	24/763 (3)	34/764 (4)	31/757 (4)	41/754 (5)	
Some college	114/763 (15)	120/764 (16)	144/757 (19)	157/754 (21)	
Baseline conditions					
Coronary artery disease, No. of participants^d^	20-30	20-30	<20	<20	.27
Cancer	210/781 (27)	198/781 (25)	181/781 (23)	150/781 (19)	.002
Lifetime smoking >100 cigarettes	251/770 (33)	231/772 (30)	248/767 (32)	253/769 (33)	.58
Lifetime alcohol use ≥1 drink	766/779 (98)	763/780 (98)	763/779 (98)	763/778 (98)	.90
Duration of personal activity tracking, median (IQR) y	5.3 (3.2-7.0)	5.4 (3.4-7.0)	5.4 (3.4-7.0)	5.6 (3.5-7.1)	.05
Daily steps, median (IQR)	8599 (6751-10 768)	8374 (6639-10 467)	8222 (6338-10 364)	8115 (6340-10 187)	.01

Abbreviations: BMI, body mass index (calculated as weight in kilograms divided by height in meters squared); PRS, polygenic risk score.

^a^ Unless otherwise indicated, data are expressed as No./total No. (%) of participants. Total numbers account for missing data. Percentages have been rounded and may not total 100.

^b^ Calculated using Kruskal-Wallis test for continuous variables and Pearson χ^2^ test for categorical variables.

^c^ Includes Asian, Black or African American, Middle Eastern or North African, Native Hawaiian or Other Pacific Islander, multiple races or ethnicities, and unknown race or ethnicity.

^d^ We report <20 or values in range because the All of Us Data and Statistics Dissemination Policy does not allow displaying exact participant counts less than 20 or participant count that would allow another count value to be derived revealing a count less than 20 to protect participants’ privacy.

### Primary Cox Proportional Hazards Regression Model

**Figure 1.**
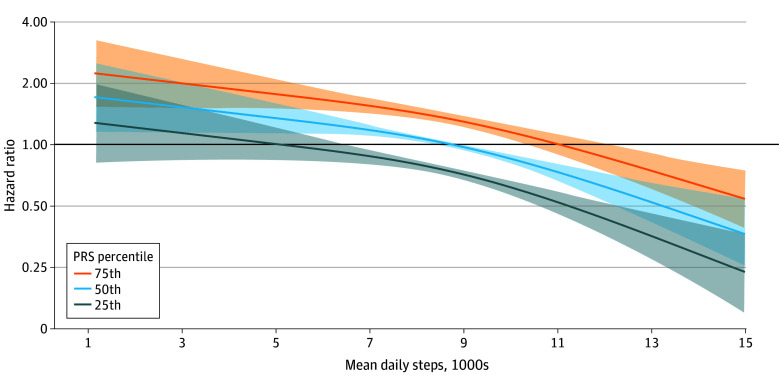
Risk of Incident Obesity Modeled by Mean Daily Step Count and Polygenic Risk Scores (PRSs) Hazard ratio for obesity was modeled according to mean daily step counts and 25th, 50th, and 75th percentile PRS for body mass index. Shaded regions represent 95% CIs. Model is adjusted for age, sex, mean baseline step counts, cancer status, coronary artery disease status, systolic blood pressure, alcohol use, educational level, and a PRS × mean steps interaction term.

**Figure 2.**
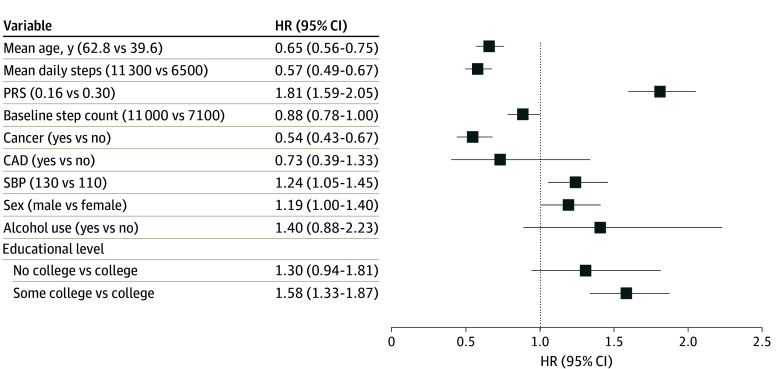
Associations Between Model Components and Hazard Ratio (HR) of Obesity Mean daily steps and polygenic risk score (PRS) for higher body mass index are independently associated with hazard for obesity. Hazard ratios model the difference between the 75th and 25th percentiles for continuous variables. CAD indicate coronary artery disease; and SBP, systolic blood pressure.

We next modeled obesity risk stratified by PRS percentile with the 50th percentile indexed to an HR for obesity of 1.00 (Figure 1). The association between PRS and incident obesity was direct (*P* = .001) and linear (chunk test for nonlinearity was nonsignificant [*P* = .07]). The PRS and mean daily step count were both independently associated with obesity risk (Figure 2). The 75th percentile BMI PRS demonstrated an 81% increase in obesity risk (HR, 1.81 [95% CI, 1.59-2.05]; *P* = 3.57 × 10^−20^) when compared with the 25th percentile BMI PRS, whereas the 75th percentile median step count demonstrated a 43% reduction in obesity risk (HR, 0.57 [95% CI, 0.49-0.67]; *P* = 5.30 × 10^−12^) when compared with the 25th percentile step count. The PRS × mean steps interaction term was not significant (χ^2^ = 1.98; *P* = .37).

Individuals with a PRS at the 75th percentile would need to walk a mean of 2280 (95% CI, 1680-3310) more steps per day (11 020 total) than those at the 50th percentile to reduce the HR for obesity to 1.00 (Figure 1). Conversely, those in the 25th percentile PRS could reach an HR of 1.00 by walking a mean of 3660 (95% CI, 2180-8740) fewer steps than those at the 50th percentile PRS. When assuming a median daily step count of 8740 (cohort median), those in the 75th percentile PRS had an HR for obesity of 1.33 (95% CI, 1.25-1.41), whereas those at the 25th percentile PRS had an obesity HR of 0.74 (95% CI, 0.69-0.79).

### Stratification by Baseline BMI

**Figure 3.**
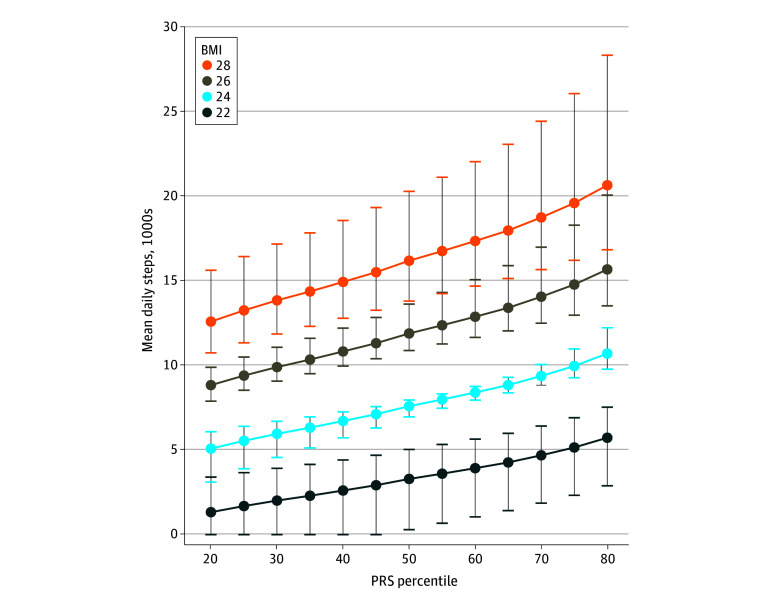
Risk of Incident Obesity Based on Mean Daily Steps and Polygenic Risk Score (PRS) Percentile Stratified by Baseline Body Mass Index (BMI) Each point estimate is indexed to a hazard ratio for obesity of 1.00 (BMI [calculated as weight in kilograms divided by height in meters squared] ≥30). Error bars represent 95% CIs.

The mean daily step count required to achieve an HR for obesity of 1.00 across the full PRS spectrum and stratified by baseline BMI is shown in Figure 3. To reach an HR of 1.00 for obesity, when stratified by baseline BMI of 22, individuals at the 50th percentile PRS would need to achieve a mean daily step count of 3290 (additional 3460 steps/d); for a baseline BMI of 24, a mean daily step count of 7590 (additional 4430 steps/d); for a baseline BMI of 26, a mean daily step count of 11 890 (additional 5380 steps/d); and for a baseline BMI of 28, a mean daily step count of 16 190 (additional 6350 steps/d).

### Sensitivity Analysis

When adding baseline BMI to the full Cox proportional hazards regression model, daily step count and BMI PRS both remain associated with obesity risk. When comparing individuals at the 75th percentile with those at the 25th percentile, the BMI PRS is associated with a 61% increased risk of obesity (HR, 1.61 [95% CI, 1.45-1.78]). Similarly, when comparing the 75th with the 25th percentiles, daily step count was associated with a 38% lower risk of obesity (HR, 0.62 [95% CI, 0.53-0.72]) (eFigure 2 in Supplement 1).

### Cumulative Incidence of Obesity

**Figure 4.**
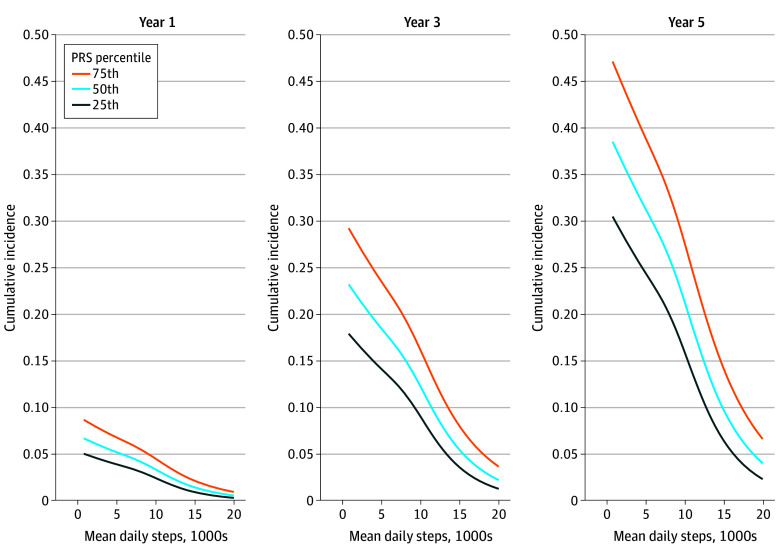
Cumulative Risk of Incident Obesity by Polygenic Risk Score (PRS) and Mean Daily Step Count at Years 1, 3, and 5

The cumulative incidence of obesity increases over time and with fewer daily steps and higher PRS. The cumulative incidence of obesity would be 2.9% at the 25th percentile, 3.9% at the 50th percentile, and 5.2% at the 75th percentile for PRS in year 1; 10.5% at the 25th percentile, 14.0% at the 50th percentile, and 18.2% at the 75th percentile for PRS in year 3; and 18.5% at the 25th percentile, 24.3% at the 50th percentile, and 30.9% at the 75th percentile for PRS in year 5 (Figure 4). The eTable in Supplement 1 models the expected cumulative incidence of obesity at 1, 3, and 5 years based on PRS and assumed mean daily steps of 7500, 10 000, and 12 500.

## Discussion

We examined the combined association of daily step counts and genetic risk for increased BMI with the incidence of obesity in a large national sample with genome sequencing and long-term activity monitoring data. Lower daily step counts and higher BMI PRS were both independently associated with increased risk of obesity. As the PRS increased, the number of daily steps associated with lower risk of obesity also increased. By combining these data sources, we derived an estimate of the daily step count needed to reduce the risk of obesity based on an individual’s genetic background. Importantly, our findings suggest that genetic risk for obesity is not deterministic but can be overcome by increasing physical activity.

Our findings align with those of prior literature^9^ indicating that engaging in physical activity can mitigate genetic obesity risk and highlight the importance of genetic background for individual health and wellness. Using the data from a large population-based sample, Li et al^9^ characterized obesity risk by genotyping 12 susceptibility loci and found that higher self-reported physical activity was associated with a 40% reduction in genetic predisposition to obesity. Our study extends these results in 2 important ways. First, we leveraged objectively measured longitudinal activity data from commercial devices to focus on physical activity prior to and leading up to a diagnosis of obesity. Second, we used a more comprehensive genomewide risk assessment in the form of a PRS. Our results indicate that daily step count recommendations to reduce obesity risk may be personalized based on an individual’s genetic background. For instance, individuals with higher genetic risk (ie, 75th percentile PRS) would need to walk a mean of 2280 more steps per day than those at the 50th percentile of genetic risk to have a comparable risk of obesity.

These results suggest that population-based recommendations that do not account for genetic background may not accurately represent the amount of physical activity needed to reduce the risk of obesity. Population-based exercise recommendations may overestimate or underestimate physical activity needs, depending on one’s genetic background. Underestimation of physical activity required to reduce obesity risk has the potential to be particularly detrimental to public health efforts to reduce weight-related morbidity. As such, integration of activity and genetic data could facilitate personalized activity recommendations that account for an individual’s genetic profile. The widespread use of wearable devices and the increasing demand for genetic information from both clinical and direct-to-consumer sources may soon permit testing the value of personalized activity recommendations. Efforts to integrate wearable devices and genomic data into the EHR further support the potential future clinical utility of merging these data sources to personalize lifestyle recommendations. Thus, our findings support the need for a prospective trial investigating the impact of tailoring step counts by genetic risk on chronic disease outcomes.

### Limitations

The most important limitation of this work is the lack of diversity and inclusion only of individuals with European ancestry. These findings will need validation in a more diverse population. Our cohort only included individuals who already owned a fitness tracking device and agreed to link their activity data to the AoURP dataset, which may not be generalizable to other populations. We cannot account for unmeasured confounding, and the potential for reverse causation still exists. We attempted to diminish the latter concern by excluding prevalent obesity and incident cases within the first 6 months of monitoring. Genetic risk was simplified to be specific to increased BMI; however, genetic risk for other cardiometabolic conditions could also inform obesity risk. Nongenetic factors that contribute to obesity risk such as dietary patterns were not available, reducing the explanatory power of the model. It is unlikely that the widespread use of drug classes targeting weight loss affects the generalizability of our results, because such drugs are rarely prescribed for obesity prevention, and our study focused on individuals who were not obese at baseline. Indeed, less than 0.5% of our cohort was exposed to a medication class targeting weight loss (phentermine, orlistat, or glucagonlike peptide-1 receptor agonists) prior to incident obesity or censoring. Finally, some fitness activity tracking devices may not capture nonambulatory activity as well as triaxial accelerometers.

## Conclusions

This cohort study used longitudinal activity data from commercial wearable devices, genome sequencing, and clinical data to support the notion that higher daily step counts can mitigate genetic risk for obesity. These results have important clinical and public health implications and may offer a novel strategy for addressing the obesity epidemic by informing activity recommendations that incorporate genetic information.

## Notes

Supplement 1. **eTable.** Cumulative Incidence Estimates of Obesity Based on Polygenic Risk Score for Body Mass Index and Mean Daily Steps at 1, 3, and 5 Years **eFigure 1.** CONSORT Diagram **eFigure 2.** Risk of Incident Obesity Modeled by Mean Daily Step Count and Polygenic Risk Scores Adjusted for Baseline Body Mass Index

Supplement 2.
Data Sharing Statement
