# Supplementary material for: Physical Activity and Incident Obesity Across the Spectrum of Genetic Risk for Obesity
Source: JAMA Netw Open. 2024 Mar 27;7(3):e243821. doi: 10.1001/jamanetworkopen.2024.3821 (PMC10973894; doi:10.1001/jamanetworkopen.2024.3821)
Supplement: Supplement 1. — eTable. Cumulative Incidence Estimates of Obesity Based on Polygenic Risk Score for Body Mass Index and Mean Daily Steps at 1, 3, and 5 Years eFigure 1. CONSORT Diagram eFigure 2. Risk of Incident Obesity Modeled by Mean Daily Step Count and Polygenic Risk Scores Adjusted for Baseline Body Mass Index [file jamanetwopen-e243821-s001.pdf]

Supplementary Online Content

Brittain EL, Han L, Annis J, et al. Physical activity and incident obesity across the spectrum of genetic risk for obesity. *JAMA Netw Open*. 2024;7(3):e243821. doi:10.1001/jamanetworkopen.2024.3821

eTable. Cumulative Incidence Estimates of Obesity Based on Polygenic Risk Score for Body Mass Index and Mean Daily Steps at 1, 3, and 5 Years

eFigure 1. CONSORT Diagram

eFigure 2. Risk of Incident Obesity Modeled by Mean Daily Step Count and Polygenic Risk Scores Adjusted for Baseline Body Mass Index

This supplementary material has been provided by the authors to give readers additional information about their work.

© 2024 Brittain EL et al. *JAMA Network Open*.

eTable. Cumulative Incidence Estimates of Obesity Based on Polygenic Risk Score for Body Mass Index and Mean Daily Steps at 1, 3, and 5 Years

	**PRS Percentile**		
	**25th**	**50th**	**75th**
**Year/Avg. Daily Step Count**	**CI %**	**CI %**	**CI %**
**Year 1**			
7,500	3.3	4.4	5.8
10,000	2.4	3.3	4.5
12,500	1.5	2.2	3.1
**Year 3**			
7,500	11.9	15.7	20.1
10,000	8.9	12.1	15.9
12,500	5.7	8.2	11.4
**Year 5**			
7,500	20.8	27	33.9
10,000	15.8	21.1	27.4
12,500	10.3	14.6	20

CI = cumulative incidence; PRS = polygenic risk score for body mass index

© 2024 Brittain EL et al. *JAMA Network Open*.

eFigure 1. CONSORT Diagram
Cohort creation based on available data sources. EHR = electronic health record, WGS = whole genome sequencing

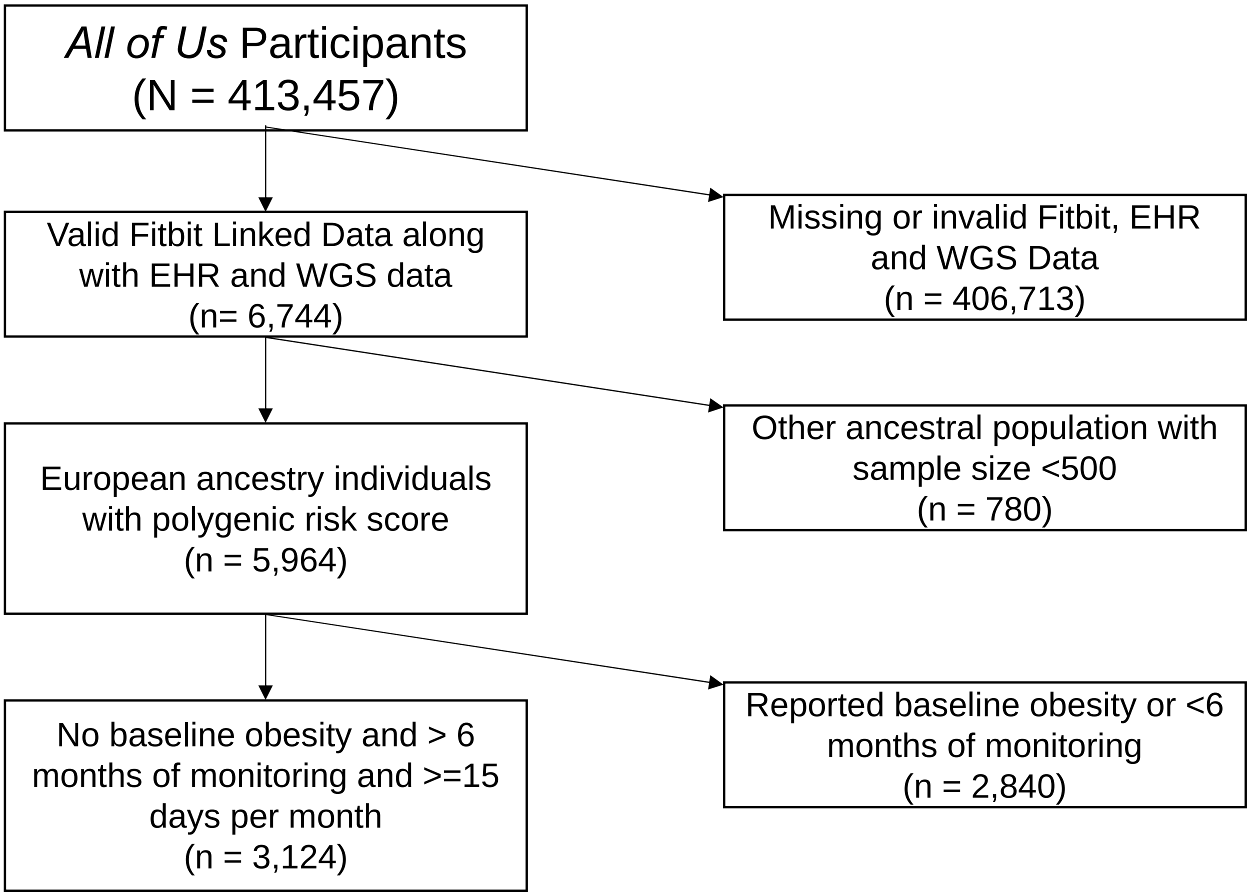

© 2024 Brittain EL et al. *JAMA Network Open*.

eFigure 2. Risk of Incident Obesity Modeled by Mean Daily Step Count and Polygenic Risk Scores Adjusted for Baseline Body Mass Index

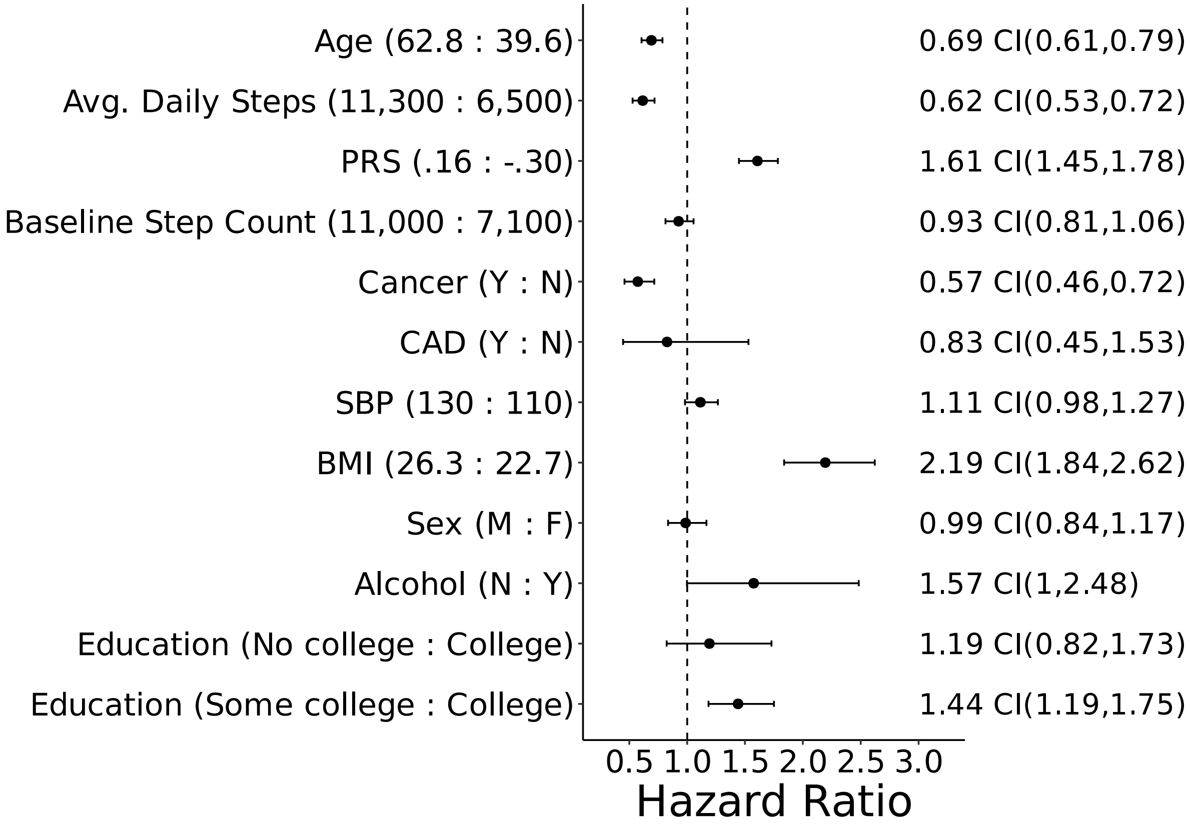

Average daily steps and polygenic risk for higher body mass index remain are independently associated with hazard for obesity when adjusting for baseline body mass index. Hazard ratio point estimates along with 95% confidence intervals are shown on the right. Hazard ratios model the difference between the 75th and 25th percentile for continuous variables.

© 2024 Brittain EL et al. *JAMA Network Open*.
