# Supplementary material for: Physical Activity and Incident Obesity Across the Spectrum of Genetic Risk for Obesity
Source: JAMA Netw Open. 2024 Mar 27;7(3):e243821. doi: 10.1001/jamanetworkopen.2024.3821 (PMC10973894; doi:10.1001/jamanetworkopen.2024.3821)
Supplement: Supplement 2. — Data Sharing Statement [file jamanetwopen-e243821-s002.pdf]

Data Sharing Statement

Brittain. Physical Activity and Incident Obesity Across the Spectrum of Genetic Risk for Obesity. *JAMA Netw Open*. Published March 27, 2024.
doi:10.1001/jamanetworkopen.2024.3821

Data
Data available: No

Additional Information
Explanation for why data not available: Unable to share patient-level data per All of Us Research Program privacy policy
